# Mobile small RNAs as mediators of stress-induced epigenomic remodeling and systemic stress adaptation

**DOI:** 10.3389/freae.2026.1827933

**Published:** 2026-05-07

**Authors:** Muhammad Daniyal Junaid, Muhammad Shahzad Iqbal, Muhammad Waqar Nasir, Muhammad Waseem Akhter

**Affiliations:** 1 Department of Biology, Faculty of Life Sciences, University of Okara, Okara, Pakistan; 2 Department of Biochemistry, University of Okara, Okara, Pakistan; 3 Jiangsu Key Laboratory of Crop Cultivation and Physiology, Agricultural College, Yangzhou University, Yangzhou, China; 4 Department of Microbiology and Molecular Genetics, University of Okara, Okara, Pakistan

**Keywords:** abiotic stress, chromatin modification, DNA methylation, epigenomic remodeling, mobile small RNAs, stress memory, transgenerational epigenetic inheritance

## Abstract

Plants rely on sophisticated long-distance communication networks to coordinate adaptive responses to changing environmental conditions. Among these, mobile small RNAs (sRNAs), including microRNAs (miRNAs) and small interfering RNAs (siRNAs), function as critical systemic signaling molecules that move between tissues through plasmodesmata and the phloem. While their roles in post-transcriptional gene regulation are well established, emerging evidence indicates that mobile sRNAs also mediate stress-induced epigenomic remodeling in recipient tissues. Specifically, 24-nt siRNAs can direct RNA-dependent DNA methylation (RdDM) and chromatin modifications at distal genomic loci, thereby altering transcriptional states, stabilizing stress-responsive gene networks, and modulating transposon activity. Such epigenetic reprogramming can extend beyond transient stress responses, contributing to stress memory and, in some cases, to transgenerational inheritance of adaptive traits. This review synthesizes current knowledge on the biogenesis, transport, and functional integration of mobile sRNAs, with special emphasis on their capacity to reshape DNA methylation landscapes and chromatin architecture during environmental stress. We further discuss experimental evidence from grafting studies, methylome profiling, and mutant analyses that support systemic epigenetic regulation mediated by sRNAs. Finally, we explore the practical applications of leveraging mobile sRNA-driven epigenomic remodeling for sustainable crop improvement, including epi-grafting and targeted epigenome engineering strategies. By positioning mobile sRNAs as mediators of systemic epigenetic plasticity, this review emphasizes their central role in coordinating plant stress adaptation in a changing climate.

## 1 Introduction

The global food production systems are under increasing pressure from the escalating frequency and severity of abiotic and biotic stressors (Lal et al., 2023). Abiotic stresses, such as drought, salinity, heat extremes, and nutrient deficiencies, along-with biotic challenges from pathogens and insect pests, pose a direct and combined threat to crop yields, ecosystem stability, and agricultural sustainability (Kopecká et al., 2023; Dixit et al., 2024). Unlike motile organisms, plants are sessile and must continuously perceive, integrate, and adapt to their immediate environment to avoid fitness costs. This evolutionary constraint has driven the evolution of sophisticated multilayered defense and acclimation mechanisms that operate at transcriptional, post-transcriptional, translational, and epigenetic levels (Haghpanah and Namdari, 2024). Beyond local responses at the stress perception site, plant survival relies on coordinated systemic responses that integrate signals across distant organs, enabling the plant to function as an integrated biological system rather than isolated tissues (Ul Hassan et al., 2022). For example, roots sensing changes in soil water or nutrient availability must signal shoots to adjust transpiration, stomatal conductance, photosynthesis, and growth allocation, while shoots reciprocally transmit carbon status and hormonal signals to roots. This long-distance communication is crucial for plant resilience and a key target for stress-tolerance research and biotechnology (Ko and Helariutta, 2017; Li et al., 2021).

For decades, plant biologists recognized classical chemical messengers, i.e., phytohormones, peptides, and metabolites as central mediators of systemic signaling (Salvi et al., 2021). However, advances in molecular genetics and high-throughput sequencing technologies have revealed a far more complex and dynamic communication network operating within plants. Among the most transformative discoveries has been the identification of mobile small RNAs (sRNAs) as key regulators of inter-organ communication (Yan, 2022; Lin et al., 2026). Small RNAs, including microRNAs (miRNAs) and small interfering RNAs (siRNAs), are short non-coding RNA molecules that regulate gene expression primarily through RNA interference (RNAi) pathways (Feng and Guang, 2024; Zhao and Zheng, 2025). While their roles in post-transcriptional gene silencing are well established, it is their ability to move between tissues and function in distal cellular contexts that has reshaped our understanding of plant systemic regulation (Zhan and Meyers, 2023). These sRNAs, synthesized in one tissue, travel via plasmodesmata and phloem to distant organs, where they modulate target gene expression and trigger systemic adaptive responses (Voinnet, 2026).

Importantly, emerging evidence shows that mobile sRNAs also mediate stable epigenetic changes. Notably, 24-nt siRNAs direct RNA-directed DNA methylation (RdDM) at homologous loci in recipient tissues, leading to *de novo* cytosine methylation and chromatin remodeling (Hamim et al., 2025). The siRNAs guide DNA methylation by interacting with ARGONAUTE4 (AGO4) and engaging DOMAINS REARRANGED METHYLTRANSFERASE 2 (DRM2), enabling methylation across CG, CHG, and CHH contexts and altering gene expression in response to environmental stimulus (Yang et al., 2023; He et al., 2024). Evidence from grafting experiments shows that siRNAs can move between tissues and induce DNA methylation changes, supporting their role in systemic epigenetic regulation (Chow and Mosher, 2023). This environmentally driven epigenomic remodeling can stabilize stress-responsive genes, control transposable elements, and modify chromatin accessibility, contributing to adaptive plasticity (Zhang et al., 2023; Kumar et al., 2024; Bashir et al., 2025). In some cases, these stress-induced epigenetic changes persist, leading to stress memory and even transgenerational inheritance (Kumar et al., 2023; Junaid et al., 2024; Prabha et al., 2025; Sintaha, 2025). Overall, mobile sRNAs act not only as signaling molecules but also as key regulators linking environmental signals to long-term chromatin reprogramming.

This review summarizes current knowledge on mobile sRNAs, focusing on their roles in long-distance signaling and environment-driven epigenetic changes during stress adaptation. It outlines their biogenesis, movement, and mechanisms of action, including their role in guiding DNA methylation and chromatin modification in distant tissues. Their key roles in nutrient regulation, drought response, and development are discussed in relation to gene regulation and epigenetic reprogramming. Evidence from grafting, mutant studies, and methylome analyses supporting systemic epigenetic regulation is also evaluated. Finally, the review highlights practical applications, including the use of mobile sRNAs in crop improvement through genome and epigenome editing (e.g., CRISPR/Cas) and grafting (Seem et al., 2024). Overall, it provides a framework for using sRNA-mediated epigenetic plasticity to develop climate-resilient and sustainable crops.

## 2 Small RNA biogenesis and regulatory function

To understand how sRNAs function as mobile regulatory signals and potential mediators of epigenomic remodeling, it is essential to explore their biogenesis and core mechanisms of action. Plant sRNAs are a highly diverse class of non-coding RNA molecules, typically ranging from 21 to 24 nucleotides in length (Baldrich et al., 2019). These molecules play crucial roles in regulating gene expression and are primarily classified into two broad categories: microRNAs (miRNAs) and small interfering RNAs (siRNAs) (Borges and Martienssen, 2015; Rogers and Chen, 2013). Despite sharing some common processing machinery, such as the RNase III enzyme Dicer, the two classes of sRNAs differ significantly in their genomic origins, precursor structures, associated ARGONAUTE proteins, and the downstream regulatory outputs. miRNAs typically originate from endogenous hairpin-shaped precursors, while siRNAs are often derived from exogenous or transposon-related RNA molecules (Baulcombe, 2004; Voinnet, 2009).

Importantly, while both miRNAs and siRNAs primarily regulate gene expression through post-transcriptional gene silencing (PTGS), certain subclasses of siRNAs, such as those involved in the RNA-directed DNA methylation (RdDM) pathway, can mediate transcriptional gene silencing (TGS) by inducing chromatin modifications, including DNA methylation (Law and Jacobsen, 2010; Matzke and Mosher, 2014). This mechanistic diversity not only shows the complex regulation of gene expression at various levels but also reveals the critical role of sRNAs in linking RNA-based signaling to stable epigenetic modifications (Zhong et al., 2010; Ha and Kim, 2014).

### 2.1 The microRNA pathway

The biogenesis of plant miRNAs begins with the transcription of MIR genes by RNA polymerase II (Pol II), which generates primary miRNA transcripts (pri-miRNAs) (Figure 1). These pri-miRNAs are characterized by a 5′ cap structure and a 3′ polyadenylated tail, typical of Pol II transcripts (Zhang et al., 2022). The pri-miRNAs then fold into characteristic stem-loop hairpin structures, which are recognized and processed in the nucleus by the RNase III enzyme DICER-LIKE 1 (DCL1). This process is assisted by several accessory proteins, including HYPONASTIC LEAVES 1 (HYL1) and SERRATE (Rogers and Chen, 2013; Fang and Qi, 2016; Kaur et al., 2023). Through a series of sequential cleavage events, a miRNA/miRNA duplex of approximately 21 nucleotides is produced. One strand of this duplex, known as the mature miRNA, is preferentially incorporated into the RNA-induced silencing complex (RISC), with the catalytic core primarily consisting of ARGONAUTE1 (AGO1) (Vaucheret, 2008).

**FIGURE 1:**
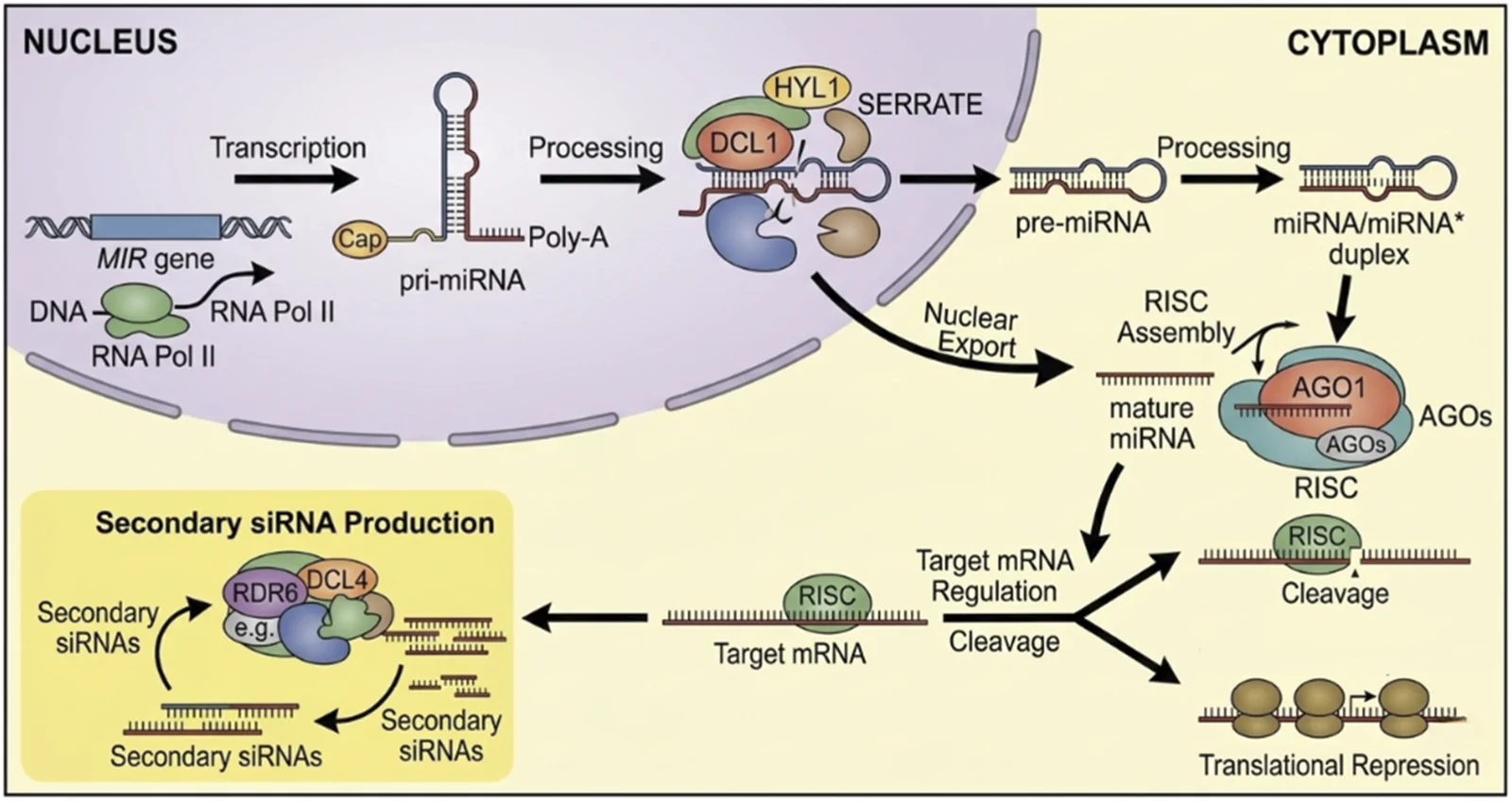
Overview of plant MicroRNA biogenesis and action.

The mature miRNA–AGO1 complex then binds to its target messenger RNAs (mRNAs) through near-perfect sequence complementarity, resulting in either endonucleolytic cleavage or translational repression of the target mRNA (Borges and Martienssen, 2015). This miRNA-mediated regulation is crucial in controlling various biological processes, including key developmental transitions, nutrient signaling, and stress-responsive gene networks. Although miRNAs predominantly mediate post-transcriptional gene silencing (PTGS), their involvement in initiating secondary siRNA production further amplifies the regulatory effects, potentially contributing to systemic and transgenerational silencing responses (Vaucheret and Voinnet, 2024).

### 2.2 The siRNA pathway and secondary amplification

In contrast to miRNAs, siRNAs are derived from long double-stranded RNA (dsRNA) precursors. These dsRNAs can originate from various sources, including exogenous factors such as viral infections, as well as endogenous sources like inverted repeats, transposable elements, or RNA-dependent RNA polymerase (RDR)-mediated conversion of single-stranded RNA into dsRNA (Borges and Martienssen, 2015; Fei et al., 2013). The biogenesis of siRNAs involves different DICER-LIKE (DCL) proteins, which produce siRNAs of various lengths, each associated with distinct regulatory outcomes (Figure 2).

**FIGURE 2:**
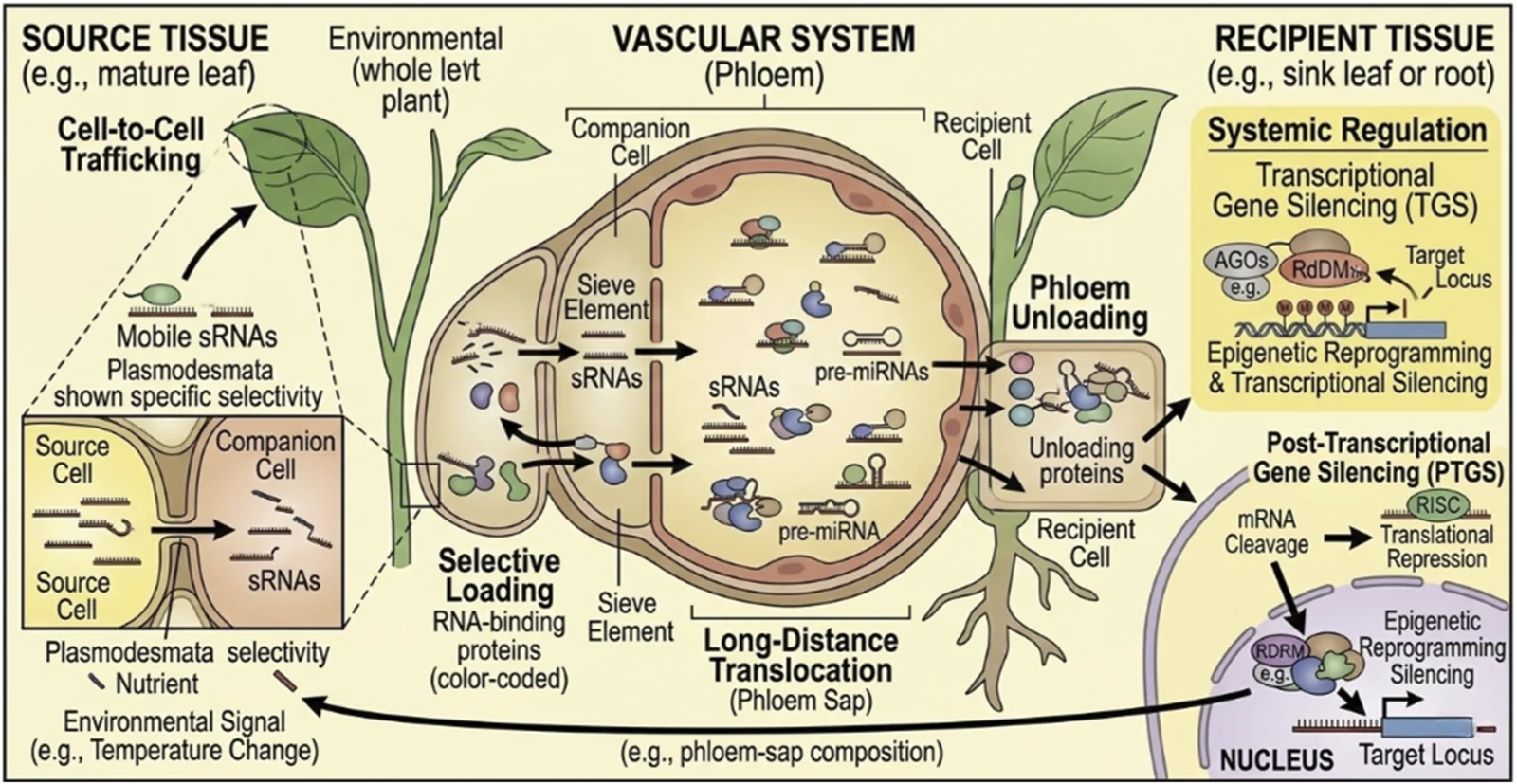
Schematic diagram of sRNA pathway and secondary amplification.

A key subclass of siRNAs involved in systemic signaling is phased secondary siRNAs (phasiRNAs), including trans-acting siRNAs (tasiRNAs). These phasiRNAs are generated when a miRNA-guided AGO complex cleaves a transcript from a target gene such as the TAS or PHAS transcript. This cleaved RNA is then converted into dsRNA by RNA-DEPENDENT RNA POLYMERASE 6 (RDR6) and processed by DCL4 or DCL2 into 21- or 22-nucleotide siRNAs in a phased register (Fei et al., 2013). This secondary amplification mechanism not only increases the abundance of these regulatory sRNAs but also enhances their ability to spread systemically, thereby amplifying the miRNA-mediated regulatory signal across tissues (Pyott and Molnar, 2015).

In addition to their involvement in post-transcriptional gene silencing (PTGS), 24-nt siRNAs produced by DCL3 play a pivotal role in transcriptional gene silencing through the RNA-directed DNA methylation (RdDM) pathway. In this pathway, RNA Polymerase IV (Pol IV) synthesizes precursor transcripts, which are then converted into dsRNA by RDR2. These dsRNAs are processed into 24-nt siRNAs by DCL3 (Matzke and Mosher, 2014; Law and Jacobsen, 2010). The siRNAs are loaded into ARGONAUTE4 (AGO4) or related AGO proteins and guide the complex to scaffold transcripts produced by RNA Polymerase V (Pol V) at target loci. This interaction facilitates the recruitment of the DNA methyltransferase DOMAINS REARRANGED METHYLTRANSFERASE 2 (DRM2), which deposits DNA methylation marks at specific genomic loci, including transposable elements, repetitive sequences, and stress-responsive genes (Law and Jacobsen, 2010; Zhang et al., 2018).

The RdDM pathway, through 24-nt siRNAs, serves as a critical mechanism for establishing or reinforcing DNA methylation at various genomic regions, ultimately modulating chromatin structure and gene expression. Furthermore, literature suggests that 24-nt siRNAs can move between tissues, inducing DNA methylation changes in recipient cells and providing a molecular basis for systemic epigenetic changes (Molnar et al., 2010; Lewsey et al., 2016). This capacity of siRNAs to mediate epigenomic reprogramming bridges environmental sensing with long-term chromatin-level regulation, thus positioning siRNA pathways as a critical link between environmental stimuli and stable transcriptional outcomes.

## 3 Mechanisms of systemic RNA mobility

Building on the role of mobile sRNAs in coordinating systemic and epigenetic responses, a key feature underlying their function is their ability to move across cellular boundaries and enter the vascular system, enabling regulation of gene expression in distant tissues. This movement is highly regulated, multi-step process involving cell-to-cell trafficking, selective phloem loading, long-distance translocation, and controlled unloading at recipient sites (Molnar et al., 2010; Pant et al., 2008). This efficient transport allows sRNAs to coordinate gene expression across tissues, supporting integrated responses to environmental changes and facilitating inter-tissue communication.

### 3.1 Cell-to-cell trafficking

The movement of sRNAs between plant cells initially occurs through plasmodesmata, specialized channels that physically connect adjacent plant cells. These channels allow small molecules, including RNA, to pass directly from one cell to another, bypassing the plasma membrane. The movement of sRNAs through plasmodesmata is tightly controlled and involves various molecular mechanisms, including the regulation of plasmodesmatal permeability and selective gating by specific protein complexes (Vaten et al., 2011; Kehr and Kragler, 2018). For example, a family of proteins known as CALLUS-SPECIFIC PROTEINS (CSPs) can influence plasmodesmata opening, thus modulating the ability of sRNAs to pass between cells (Oparka et al., 1999). Notably, the movement of sRNAs is often facilitated by the association of the RNA with specific RNA-binding proteins or other factors that promote its trafficking through plasmodesmata.

Importantly, stress conditions such as pathogen attack or abiotic perturbation can alter plasmodesmal conductivity, thereby influencing the intercellular spread of RNA silencing signals (Sager and Lee, 2014). This dynamic gating mechanism ensures that sRNA movement is tightly controlled and context-dependent, allowing plants to restrict or enhance systemic signaling as required. Such regulation provides a mechanistic foundation for how localized stress perception can initiate a controlled wave of RNA-based signaling toward the vasculature.

### 3.2 Selective phloem loading and long-distance translocation

For long-distance distribution, sRNAs must enter the sieve element–companion cell (SE–CC) complex of the phloem. Sieve elements (SEs) are enucleate and metabolically dependent on adjacent companion cells (CCs), which provide proteins, RNAs, and metabolic support (Kehr and Kragler, 2018). Small RNAs synthesized in companion cells can move into sieve elements through specialized, highly branched plasmodesmata connecting the two cell types (Lucas et al., 2009).

Once loaded into the sieve element, sRNAs are transported through the phloem stream via pressure-driven mass flow, moving from source tissues (typically mature leaves) to sink tissues such as roots, developing meristems, and reproductive organs (Kehr and Kragler, 2018). Grafting experiments have provided compelling evidence that endogenous sRNAs including 24-nt siRNAs can travel across graft junctions and accumulate in distal tissues, where they retain functional activity (Molnar et al., 2010; Lewsey et al., 2016). In some cases, mobile siRNAs arriving in recipient tissues have been shown to direct DNA methylation at homologous loci, linking phloem-mediated transport to systemic epigenomic remodeling. Importantly, the process of long-distance translocation is dynamic and regulated, with the movement of specific sRNAs often being controlled by developmental cues or in response to environmental stressors such as drought, pathogen infection, or herbivory (Pant et al., 2008; Molnar et al., 2010).

Phloem-loading is typically regulated by a variety of transporters, including SWEET transporters and other membrane-associated proteins, which are capable of recognizing specific sRNA molecules (Chen et al., 2012). This ensures that only specific, functionally relevant sRNAs are transported through the plant, preventing the movement of nonspecific or harmful RNAs.

### 3.3 Controlled unloading in recipient organs

Upon reaching distant tissues, sRNAs must be unloaded from the phloem and delivered into the recipient cells, where they exert their regulatory functions. The unloading of sRNAs is a complex process, often involving the recognition of specific receptors or transporters that enable the sRNAs to cross the cell membrane into the cytoplasm of the recipient cells (Sager and Lee, 2014). This unloading mechanism is not passive; rather, it is actively regulated to ensure that sRNAs are only delivered to tissues where their regulatory functions are required. In some cases, sRNAs are reloaded into new transport systems to move between additional tissues, enabling the establishment of systemic responses throughout the plant (Lin et al., 2026).

### 3.4 Role in transcriptional and epigenetic reprogramming

The systemic mobility of sRNAs is essential for coordinating whole-plant responses to environmental stimuli. By transmitting small RNA signals across tissues, plants can achieve synchronized changes in gene expression, including the regulation of stress-responsive genes, developmental pathways, and metabolic processes (Shivaprasad et al., 2012). In addition to post-transcriptional gene silencing (PTGS), mobile sRNAs, such as those involved in RNA-directed DNA methylation (RdDM), can also induce transcriptional and epigenetic reprogramming. Through these mechanisms, sRNAs contribute to establishing stable, long-lasting changes in gene expression that allow the plant to adapt to environmental challenges (Molnar et al., 2010; Lewsey et al., 2016). This systemic regulation links environmental perception to stable changes in plant growth, development, and stress resilience (Seem et al., 2026).

### 3.5 Protection and stability: RNA-binding proteins and ribonucleoprotein complexes

The phloem sap contains ribonucleases that could potentially degrade unprotected RNA molecules. Therefore, mobile sRNAs are thought to be stabilized during transit through association with RNA-binding proteins (RBPs), forming ribonucleoprotein (RNP) complexes. One of the earliest identified phloem-associated RBPs, PHLOEM SMALL RNA-BINDING PROTEIN 1 (PSRP1), was shown to bind single-stranded small RNAs and is proposed to facilitate their stability and mobility (Yoo et al., 2004). Such RNP complexes may protect sRNAs from degradation while also contributing to their selective transport and delivery.

In addition to RNP complexes, increasing evidence suggests that extracellular vesicle-like structures may participate in RNA transport in plants, particularly in inter-organismal communication during plant–pathogen interactions (Cai et al., 2018). While the precise contribution of vesicle-mediated transport to long-distance systemic mobility within plants remains under active investigation, these findings support the broader concept that plants utilize protective packaging mechanisms to preserve the integrity and regulatory potential of mobile sRNAs.

### 3.6 The mobile RNAome and coordinated regulatory outputs

The phloem sap contains a diverse population of mobile RNAs including messenger RNAs (mRNAs), transfer RNAs (tRNAs), long non-coding RNAs (lncRNAs), and small RNAs collectively referred to as the mobile RNAome (Kehr and Kragler, 2018). This molecular diversity suggests a hierarchical and integrated regulatory system rather than isolated signal transmission. High-throughput analyses have demonstrated that specific transcripts and sRNAs are selectively enriched in phloem sap, indicating regulated loading rather than stochastic leakage (Pant et al., 2008).

The co-transport of mRNAs and regulatory sRNAs provides an elegant mechanism for fine-tuning gene expression in recipient tissues. In this model, a transcript encoding a stress-responsive protein may be transported together with an sRNA capable of modulating its abundance, thereby acting as a regulatory “dimmer switch” that calibrates expression upon arrival. Furthermore, when mobile 24-nt siRNAs reach sink tissues, they may initiate local amplification or engage the RNA-directed DNA methylation machinery, reinforcing transcriptional silencing and potentially stabilizing stress-adaptive gene expression patterns (Molnar et al., 2010; Lewsey et al., 2016; Law and Jacobsen, 2010). Through this integrated network of RNA mobility, protection, and regulatory targeting, plants achieve precise and context-dependent systemic coordination that can extend from rapid post-transcriptional responses to durable chromatin-level modifications (Figure 3).

**FIGURE 3:**
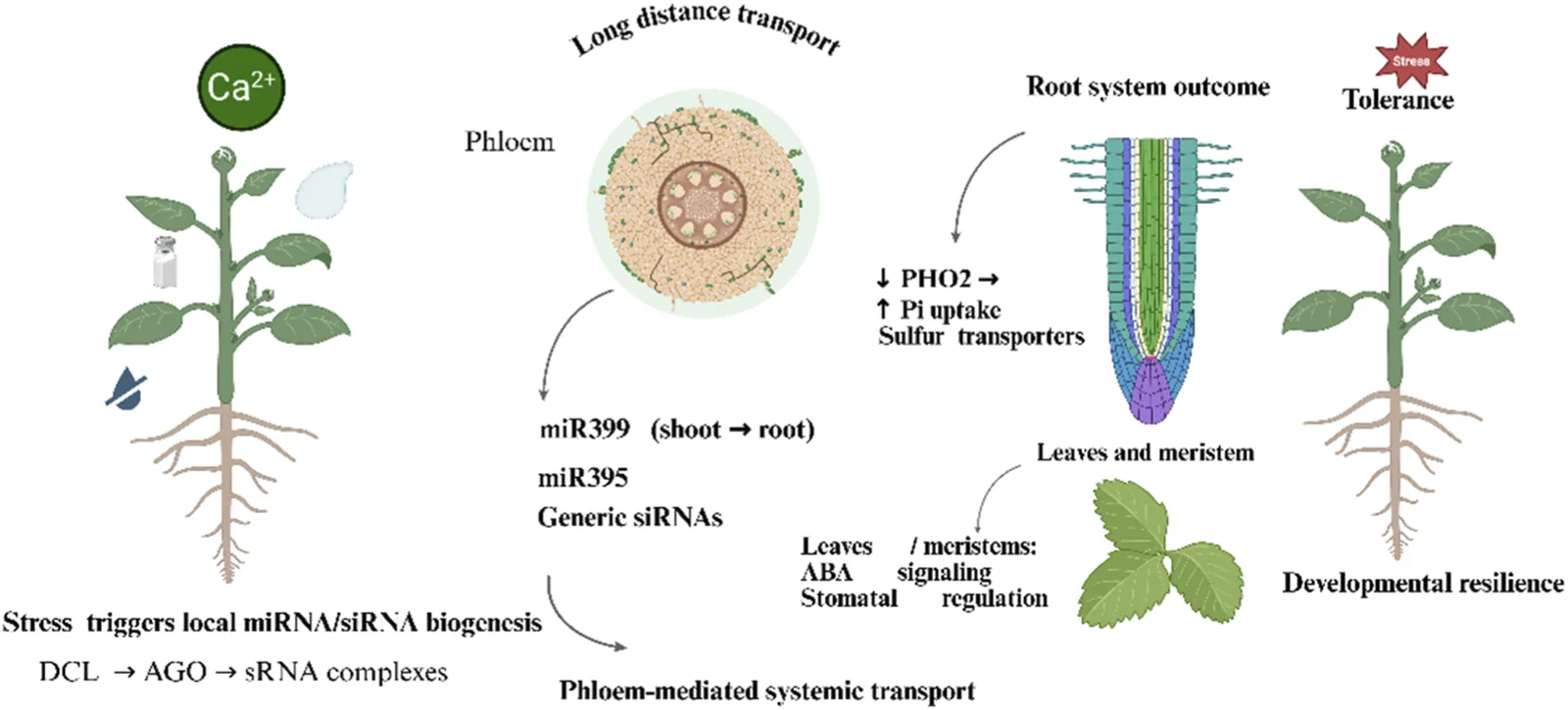
Mobile sRNAs integrate environmental signals with whole-plant responses.

## 4 Mobile sRNAs in systemic stress adaptation

Abiotic stresses, including drought, temperature extremes, and nutrient limitation are among the most severe constraints on global crop productivity. Because stress perception is often spatially restricted (e.g., roots sensing water or nutrient availability), plant survival depends on the rapid transmission of information to distal organs. Accumulating evidence demonstrates that mobile sRNAs, particularly miRNAs, function as key components of these systemic signaling networks by coordinating transcriptional and post-transcriptional responses across the whole plant (Table 1).

**TABLE 1:** Mobile small RNAs involved in systemic signaling in plants.

sRNA	Primary stress/Process	Transport direction (typical)	Level of validation	Known/Proposed targets	Translational potential	References
miR399	Phosphate starvation	Shoot → root	High	*PHO2* (E2 ubiquitin-conjugating enzyme)	High (conceptual)	Pant et al. (2008)
miR395	Sulfur deficiency	Shoot → root	Medium–high	*SULTR1;2*, *APS* family genes	Medium–high	Yang et al. (2025)
miR169	Drought, nitrogen status	Root → shoot (proposed; context-dependent)	Medium	NF-YA transcription factor family	Medium	Ni et al. (2013)
miR165/166	Vascular development, root plasticity	Local (cell-to-cell)	High (local)/Low (systemic)	Class III HD-ZIP transcription factors	Low (systemic)	Sakaguchi and Watanabe (2012)
miR156	Developmental phase transition	Leaf → SAM	Medium–high	*SPL* transcription factors	Low–medium	Jerome Jeyakumar et al. (2020)
phasiRNAs/tasiARFs	Development, stress crosstalk	Putative systemic (species-dependent)	Low–medium	*ARF* family genes	Low	Valdés-López et al. (2019)

### 4.1 Systemic transport of mobile sRNAs

A key property of mobile sRNAs is their ability to move beyond their cells of origin and regulate gene expression in distant tissues. After biogenesis, selected sRNAs are incorporated into ribonucleoprotein complexes and transported through symplastic pathways (Tang et al., 2022). Their short-range movement occurs via plasmodesmata, while long-distance transport is primarily mediated by the phloem, which serves as the plant’s systemic communication network (Zambryski and Crawford, 2000). sRNA movement is bidirectional: shoot-derived sRNAs can travel to roots to regulate nutrient uptake and root development, whereas root-derived sRNAs move to shoots to transfer information about soil conditions and stresses (Lin et al., 2026). This two-way signaling enables coordinated whole-plant responses. Importantly, sRNA transport is selective and regulated rather than passive. Only specific sRNAs are efficiently loaded into the phloem, often with RNA-binding proteins or extracellular vesicles that enhance stability and mobility. Evidence from grafting and transgenic studies shows that mobile sRNAs can cross tissue boundaries and alter gene expression in distant organs. For example, in walnut, sRNAs produced in transformed rootstocks targeting JrGA20ox1 were transported to wild-type scions, where they modulated hormone signaling and drought responses (Lou et al., 2024).

### 4.2 Drought stress and osmotic regulation

Drought stress initiates in the root system, where reduced soil water availability is sensed and converted into long-distance signals that regulate shoot physiology, most notably stomatal conductance. In addition to classical hormonal signals such as abscisic acid (ABA), mobile sRNAs have emerged as important contributors to this root-to-shoot communication.

### 4.3 miR169 and ABA-dependent signaling

miR169 represents one of the best-characterized examples of an sRNA implicated in systemic drought responses (Figure 4). Under water deficit conditions, miR169 levels are differentially regulated across tissues, with evidence indicating reduced expression in roots but enrichment in the phloem sap and aerial tissues (Lei et al., 2025). In leaves, miR169 targets members of the Nuclear Factor Y, subunit A (NF-YA) transcription factor family, which play key roles in ABA-mediated stomatal regulation (Chakraborty et al., 2025; Seem et al., 2025a). Although the precise dynamics of miR169 mobility remain under investigation, its systemic accumulation correlates with enhanced stomatal closure and improved water-use efficiency, supporting a functional role in drought adaptation.

**FIGURE 4:**
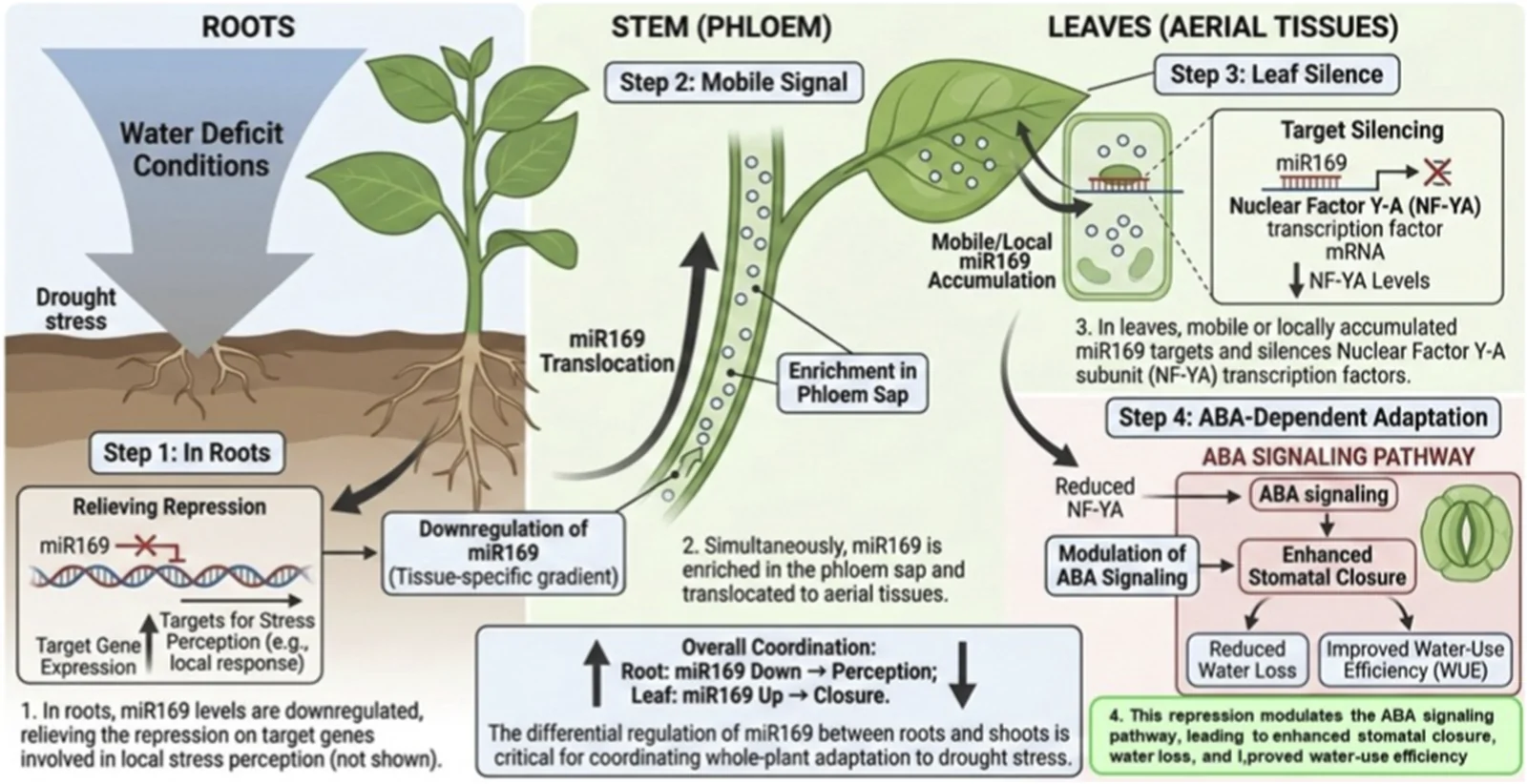
Systemic role of miR169 in ABA-dependent drought adaptation.

### 4.4 miR165/166 and root hydraulic plasticity

In contrast to long-distance root-to-shoot signaling, miR165 and miR166 primarily mediate short-range intercellular movement within root tissues. These miRNAs move from the endodermis into the stele, where they establish gradients of Class III HD-ZIP transcription factors that determine xylem patterning and vascular differentiation (Miyashima et al., 2011; Singh et al., 2014). By modulating vascular architecture, this local mobility indirectly contributes to systemic drought tolerance by optimizing water transport capacity. Together, these examples illustrate how both local and long-distance sRNA movement contribute to coordinated osmotic regulation.

### 4.5 Nutrient deficiency and resource allocation

Efficient nutrient acquisition requires continuous communication between source tissues (shoots) and sink tissues (roots). Mobile sRNAs function as molecular indicators of nutrient status, enabling plants to dynamically adjust uptake, transport, and allocation strategies.

### 4.6 Phosphate starvation and miR399

The shoot-to-root transport of miR399 during phosphate (Pi) deficiency is the most definitive example of systemic sRNA signaling in plants. Under low Pi conditions, miR399 is strongly induced in leaves and translocated via the phloem to the roots (Pant et al., 2008). In root cells, miR399 targets PHO2, which encodes an E2 ubiquitin-conjugating enzyme that negatively regulates phosphate transporters. Silencing of PHO2 relieves this repression, resulting in enhanced Pi uptake and translocation to the shoot (Kumar et al., 2021). This pathway exemplifies a highly specific and experimentally validated long-distance regulatory circuit that aligns nutrient acquisition with shoot demand.

### 4.7 Sulfur and nitrogen signaling

A comparable mechanism operates during sulfur limitation, where mobile miR395 regulates sulfate transport by targeting SULTR family genes, including SULTR1; 2 (Kawashima et al., 2009; Rauf et al., 2024). In grafted systems, miR395 has been shown to move across the graft junction, highlighting the robustness of systemic sRNA transport pathways (Figure 5).

**FIGURE 5:**
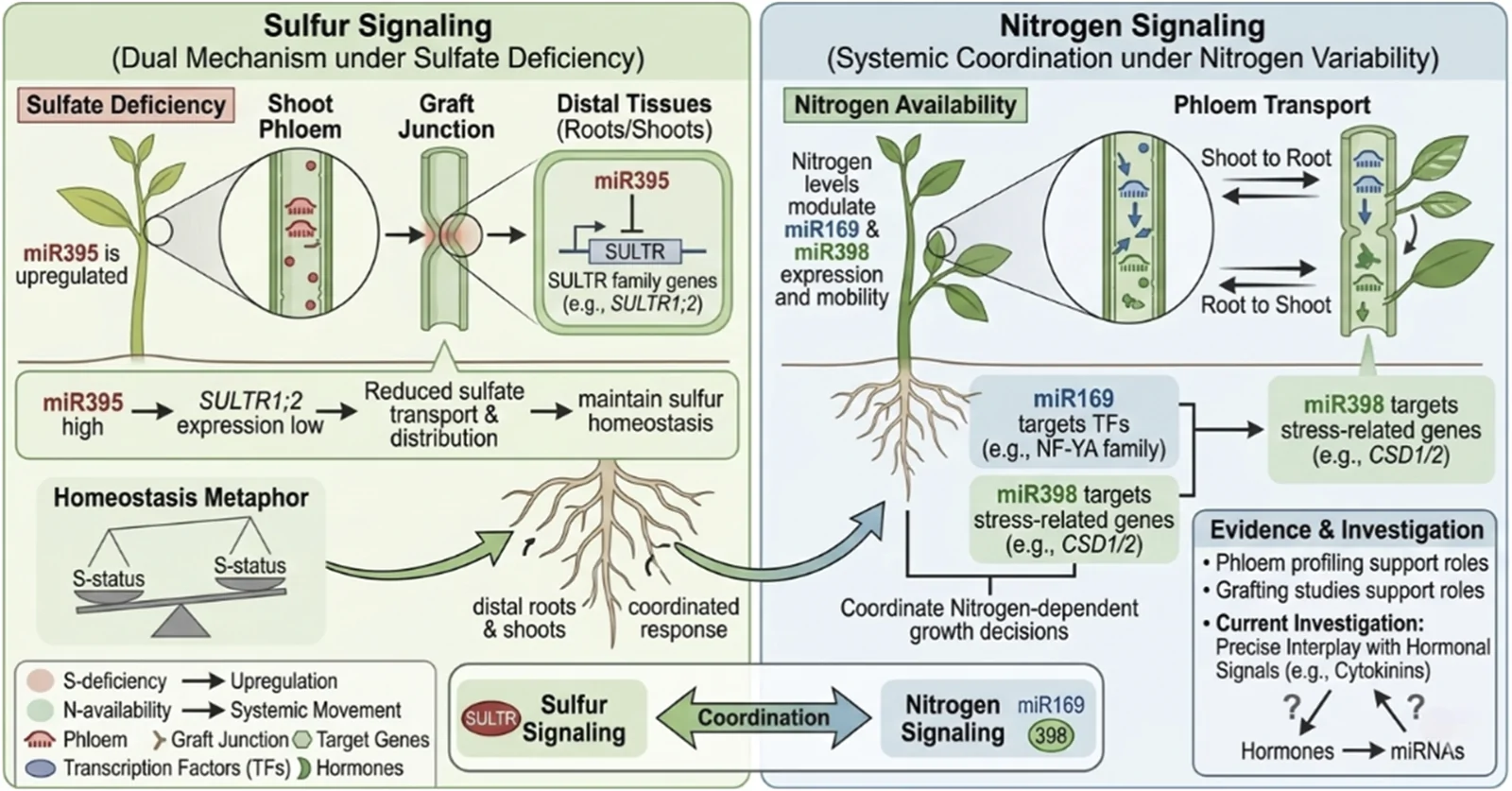
Schematic diagram of miRNA signaling networks coordinate sulfur and nitrogen homeostasis.

Nitrogen availability also influences systemic miRNA signaling, with miR169 and miR398 implicated in coordinating nitrogen-dependent growth decisions between roots and shoots (Xu et al., 2011). While the mobility of these miRNAs is supported by grafting and phloem profiling studies, their precise transport routes and quantitative contribution relative to hormonal signals remain active areas of research.

### 4.8 Integration of abiotic stress signals at the whole-plant level

Systemic sRNA transport is tightly regulated and context-dependent, rather than constitutive or indiscriminate. Different stress conditions induce distinct sRNA populations, which are selectively mobilized to appropriate target tissues. In the case of drought and salinity stress, grafting experiments provide indirect but compelling evidence for mobile RNA-mediated signaling. Drought-tolerant rootstocks can confer enhanced stress tolerance to sensitive scions, an effect proposed to involve the transfer of mobile sRNAs alongside hormones and peptides (Zafar et al., 2024). The prime candidates for these graft-transmissible signals are sRNAs, as they possess the unique capacity for sequence-specific epigenetic regulation. Unlike hormones which trigger general responses, a mobile sRNA can silence specific target genes in the scion, providing a precise molecular memory of the stress experienced by the rootstock. Although the identity and relative contribution of individual sRNAs in these systems are still being resolved, these observations underscore the functional importance of systemic RNA mobility in environmental adaptation.

### 4.9 A general-purpose systemic sRNA network

The transport system that carries stress-responsive sRNAs is not stress-specific; the same phloem-based network also supports developmental regulation, indicating a general-purpose communication system in plants (van Bel et al., 2013). This system relies on phloem companion cells, which selectively load sRNAs into sieve tubes for delivery to distant tissues. For instance, miR156 moves from leaves to the shoot apical meristem to control flowering by repressing SPL transcription factors (Poethig, 2013), while miR167 regulates adventitious root formation in recipient tissues (Rauf et al., 2024). Together, these findings suggest that mobile sRNAs function as versatile signals integrating environmental, nutritional, and developmental cues through a shared transport network. Because this system operates independently of signal origin, it enables plants to coordinate growth, defense, and resource allocation, providing flexibility under changing conditions (Li et al., 2024).

## 5 Translational applications of mobile sRNAs in crop improvement

The recognition that sRNAs act as systemic signaling molecules has expanded their role from local regulators to key coordinators of whole-plant responses. By enabling communication between distant tissues, mobile sRNAs allow plants to integrate environmental cues such as nutrient limitation, drought, salinity, and pathogen attack into coordinated physiological responses. This systemic regulatory layer presents new opportunities for crop improvement, particularly under field conditions where stresses are spatially and temporally variable. Targeting sRNA mobility or responsiveness may therefore enhance plant adaptability (Kumar and Mohapatra, 2021; Zhan and Meyers, 2023; Kaur et al., 2024; Lin et al., 2026). Naturally occurring mobile miRNAs illustrate this potential. For example, miR399 moves from shoots to roots under phosphate deficiency to repress PHO2 and enhance phosphate uptake, while miR395 regulates sulfur homeostasis through long-distance signaling (Pant et al., 2008). These systems demonstrate how localized sRNA production can generate widespread physiological effects.

A key advantage of mobile sRNA pathways is their ability to amplify regulatory signals. Primary sRNA activity can trigger secondary siRNA production or RNA-directed DNA methylation (RdDM). This amplification enables local stress perception to affect distant tissues and may also contribute to stress memory through stable epigenetic modifications, with potential transgenerational effects (Tamiru et al., 2018; Erdmann and Picard, 2020; Seem et al., 2025a).

From a translational perspective, four main strategies can be used to exploit mobile sRNAs: (i) genome editing of endogenous sRNA pathways, (ii) synthetic sRNA design, (iii) targeted epigenome modification, and (iv) graft-based delivery systems. Genome editing using CRISPR/Cas can refine MIR gene expression or target-site sensitivity, enable precise and context-dependent regulation while minimizing pleiotropic effects (Ben-Laouane et al., 2026; Meenakshi et al., 2026). Synthetic approaches, including artificial miRNAs and ta-siRNA systems, can enhance specificity and regulatory strength, though careful design is required to avoid off-target effects (Miloro et al., 2024; Gahoi and Gautam, 2025). Epigenome-targeting strategies further expand these possibilities by using sRNA-guided RdDM to establish stable transcriptional states without altering DNA sequences. While this approach offers potential for durable trait regulation and stress memory, the stability and heritability of induced epigenetic changes require further validation (Ali and Tang, 2025).

Grafting provides a practical, non-transgenic strategy for deploying mobile sRNAs, as engineered rootstocks can transmit regulatory signals to non-modified scions. This approach is particularly promising in horticultural systems but depends on graft compatibility and efficient RNA movement (Kapazoglou et al., 2021; Tsaballa et al., 2021). In parallel, non-genetic delivery methods, such as nanoparticle-based RNA applications, are being explored, although their systemic mobility remains variable (Mat Jalaluddin et al., 2023; Xiao et al., 2025). Despite these advances, several challenges remain. Mobile sRNA pathways are closely integrated with developmental and hormonal networks, increasing the risk of unintended effects. Precise dosage control and comprehensive molecular analyses are essential. In addition, species-specific differences in sRNA mobility and transport mechanisms may limit direct translation from model systems to crops (Xiang et al., 2023; Dayal et al., 2024). A structured development pipeline combining target identification, mechanistic validation, field testing, and biosafety assessment will be critical for practical application. Overall, mobile sRNAs offer a shift from gene-level modification to the engineering of systemic regulatory networks, providing a promising framework for developing resilient crops in the face of climate change (Ahmad et al., 2025).

### 5.1 Field translation, biosafety, and regulatory considerations

Successful application of mobile sRNAs in crop improvement requires validation under real agricultural conditions. Moving from controlled environments to the field introduces variability in climate, soil, and biotic interactions, which can affect the stability of sRNA-mediated traits. Thus, beyond molecular design, practical deployment depends on consistent agronomic performance, biosafety, and regulatory acceptance. A major challenge is maintaining stable systemic sRNA signaling. Long-distance movement depends on efficient phloem loading, RNA stability, and responsiveness of recipient tissues all of which are influenced by environmental factors such as temperature, nutrients, and stress conditions. Multi-location field trials are therefore essential to confirm consistent performance. In addition, careful dosage control is required, as excessive silencing may negatively affect growth under non-stress conditions (Kehr and Kragler, 2018; Kehr et al., 2022).

Pleiotropic effects also need to be considered, since many mobile miRNAs regulate both development and stress responses. Mis-regulation can alter traits such as flowering time or yield. Comprehensive field evaluations, supported by transcriptomic and methylomic analyses, are necessary to detect unintended effects (Erdmann and Picard, 2020; Schaefer, 2021).

Biosafety is particularly relevant for approaches involving epigenetic modification or secondary siRNA amplification. Off-target DNA methylation is a potential risk, especially in repetitive genomes. Genome-wide analyses, including methylome and sRNA profiling, are required to ensure specificity and assess the stability and heritability of induced changes (Gallusci et al., 2023). Ecological impacts must also be evaluated, as mobile sRNAs can influence nutrient cycling and plant interactions with microbes, pests, and beneficial organisms. Risk assessment should therefore include soil microbiome dynamics and potential effects on non-target species (Pant et al., 2008; Arpaia et al., 2017). Regulatory approval will depend on the method used. Genome editing, transgenic approaches, and graft-based systems may be treated differently across jurisdictions. In particular, grafting strategies where only the rootstock is engineered may offer advantages if the harvested tissues remain transgene-free, although molecular validation is still required (Li et al., 2026).

Practical adoption depends on scalability, cost, and compatibility with cropping systems. Genome editing may suit annual crops, whereas graft-based approaches are more applicable to perennials. Economic benefits, such as reduced fertilizer use through improved nutrient signaling, should also be considered (Simoni et al., 2025). Overall, translating mobile sRNA technologies to agriculture requires integrating molecular precision with field-level performance. Coordinated efforts across molecular biology, agronomy, and regulatory science will be essential to develop resilient crops capable of stable performance under variable environmental conditions (Saini and Kole, 2024; Wagh et al., 2026). A conceptual overview of these strategies is presented in Figure 6.

**FIGURE 6:**
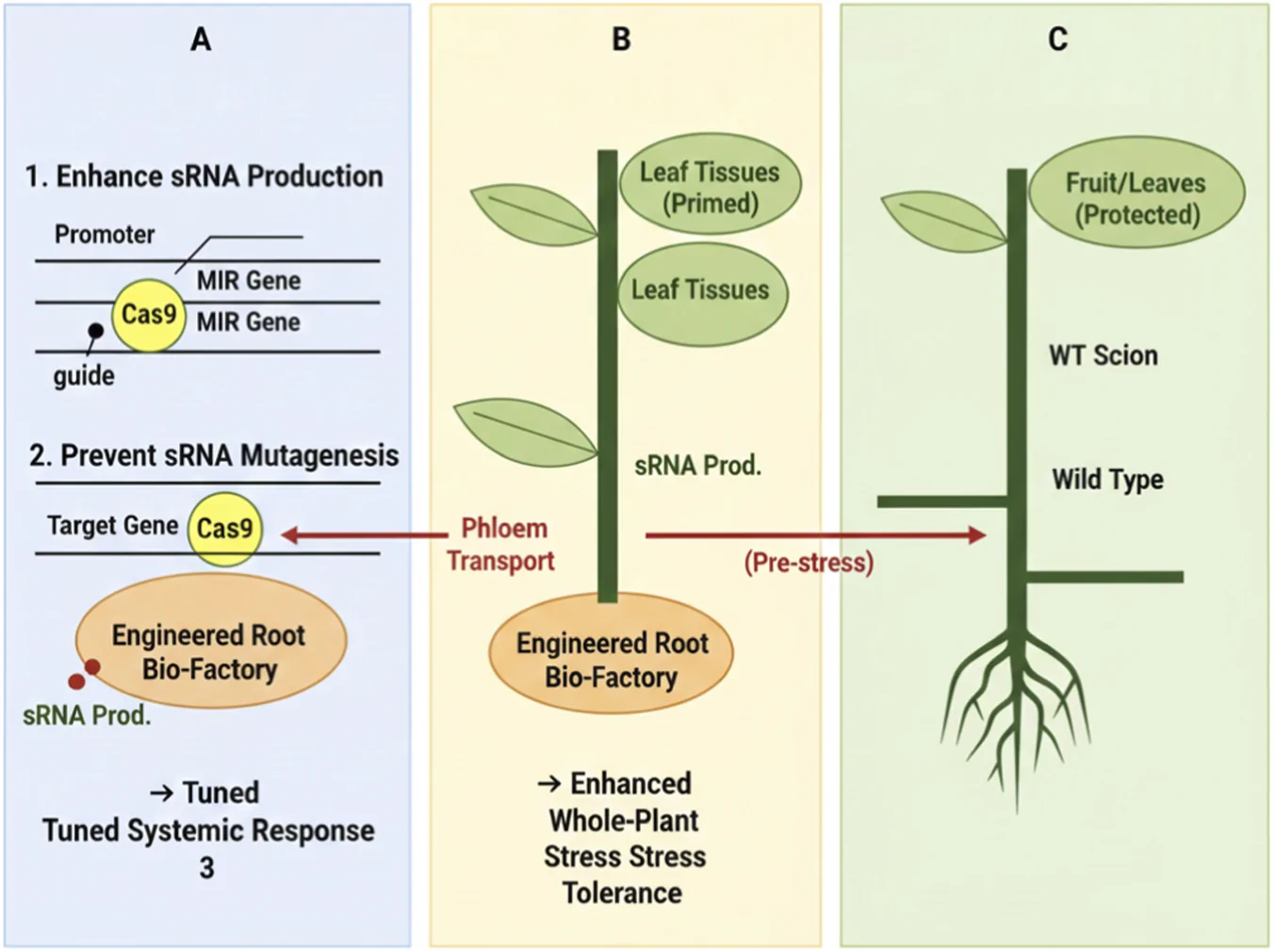
Bioengineering strategies for exploiting mobile small RNAs in crop improvement. **(A)** GENE EDITING (CRISP/Cas9) **(B)** “PRIMED” PLANT (Tissue-Specific Bio-Factory) **(C)** TRANSGENIC GRAFTING (Rootstock-to-Scion Signaling)

### 5.2 Network complexity and the pleiotropy paradox

A central paradox in translating mobile sRNA biology is that the same properties that make these molecules powerful systemic regulators broad mobility and integration into hormonal and developmental circuits also create a high risk of pleiotropy. Because phloem-mobile sRNAs routinely participate in both stress-responsive and developmental programs, manipulating a single mobile sRNA to enhance tolerance to drought, salinity, or nutrient limitation can unintentionally perturb flowering time, meristem identity, or biomass allocation (Lucas et al., 2013; Zhao et al., 2025). That trade-off is not a failure of tactics but an intrinsic property of networked signaling: coordination at the whole-plant scale necessarily couples multiple traits.

The redundancy and buffering built into systemic signaling further complicate prediction. Mobile sRNAs do not act in isolation; they interface with phytohormone gradients, transcription factor cascades, peptide signals, metabolites, mobile proteins, and chromatin-based regulation, forming circuits with feedback, feed-forward, and cross-regulatory motifs (Yan and Ham, 2022; Dawar et al., 2025). Perturbation of a single node can be hindered by compensatory pathways, produce context-dependent responses, or trigger secondary siRNA amplification and RdDM at homologous loci mechanisms that can broaden the downstream effects of an initial intervention. Practically, this means single-gene or single-sRNA edits are often insufficient to produce robust, field-relevant phenotypes.

The engineering implication is straightforward: interventions must be designed with network architecture in mind. Spatial restriction (tissue-specific or stress-inducible promoters), quantitative tuning of target-site complementarity, and modular combinatorial edits are strategies to reduce pleiotropy while retaining systemic efficacy. Crucially, these refinements require mechanistic knowledge of how sRNA signals propagate and are interpreted across organs and developmental stages. Without that dynamic understanding, even well-intentioned manipulations risk unintended amplification of secondary pathways or stable epigenomic shifts that persist beyond the intended context.

### 5.3 Technical and applied constraints

Beyond biological complexity, translational deployment faces several practical constraints. Grafting is a proven route for long-distance signal transfer, but its scalability and species-specific applicability vary rootstock engineering is realistic for many perennial and horticultural systems but impractical at scale for annual cereals and many legumes, where genome editing or other genetic strategies will remain necessary.

Exogenous delivery of synthetic sRNAs faces physicochemical and logistical barriers. Naked RNA is vulnerable to nucleases, UV, and microbial degradation; cuticular and root uptake are variable; and systemic redistribution after topical application is often unpredictable (Kumar, 2025). Nanoparticle carriers, clay nanosheets, and other stabilization chemistries hold promise, but controlled release, target specificity, cost, and regulatory acceptance remain unresolved (Venu et al., 2025; Bania et al., 2026). Until delivery platforms reliably produce predictable systemic mobility and tissue targeting, genetic or graft-based approaches will likely offer the most dependable translational path.

Dose and timing are additional technical bottlenecks. Mobile sRNA activity is concentration- and time-dependent: under-delivery may fail to trigger adaptive responses, whereas over-accumulation can saturate ARGONAUTE complexes, elicit off-target secondary siRNA production, or promote unintended RdDM and persistent epigenetic change (Lucas et al., 2013; Voinnet, 2026). Quantifying the kinetics of sRNA biogenesis, phloem loading, transport, unloading, and degradation under field conditions therefore become essential. Addressing these gaps will require quantitative tools phloem sap profiling, high-resolution imaging, and mathematical models of RNA flux coupled with field-scale validation.

### 5.4 Toward a systems-level framework for predictive translation

Progress requires moving from descriptive catalogs of mobile sRNAs to an integrative, predictive framework that treats the phloem as a dynamic “mobile-ome” where sRNAs, proteins, peptides, metabolites, hormones, and chromatin signals coalesce (van Bel et al., 2013; Kehr et al., 2022). High-resolution, spatially resolved profiling of phloem sap and recipient tissues across developmental stages and environmental gradients will be central to this effort.

Integrative ‘omics small RNA-seq, transcriptomics, proteomics, metabolomics, and methylome mapping must be combined with temporal sampling and precise phenotyping to identify regulatory hubs, dominant signal hierarchies, and nodes with favorable benefit-to-pleiotropy ratios. Computational systems biology and machine learning can then translate these multi-layer datasets into models that predict systemic signal propagation and prioritize candidate sRNAs or network interventions with maximal agronomic value and minimal collateral effects. Importantly, these models should explicitly incorporate epigenomic consequences (e.g., potential for RdDM and heritable chromatin changes) so that predictions account for both immediate physiological outcomes and longer-term stability or transgenerational effects.

Ultimately, a predictive translational pipeline will integrate mechanistic genetics, quantitative physiology, systems modeling, and field agronomy. This network-level design ethos, grounded in rigorous profiling and modeling can transform mobile sRNA engineering from an empirical exercise into a rational strategy for producing crops that achieve coordinated, durable stress adaptation while minimizing pleiotropic and ecological risks.

## 6 Conclusion and future perspectives

Plants employ sophisticated long-distance communication networks to drive adaptive strategies in response to fluctuating environmental stimuli. Emerging research highlights the pivotal role of mobile small RNAs (sRNAs) within these systems, bridging localized sensing of environmental changes with genome-wide regulatory adjustments across the organism. These sRNAs, including miRNAs and siRNAs, stretch across plasmodesmata and the phloem vasculature to modulate gene expression in remote cells and tissues. Notably, 24-nucleotide siRNAs facilitate RNA-directed DNA methylation (RdDM), allowing mobile sRNAs to propagate epigenetic modifications and alter chromatin architecture far from their production sites. These processes not only drive acute stress mitigation but also support durable adaptations, potentially contributing to transgenerational stress priming and memory. Cutting-edge methodologies, such as high-throughput sRNA sequencing, heterografting experiments, and genome-wide methylation profiling, have significantly expanded our insights into systemic RNA dynamics. These studies reveal how mobile sRNAs harmonize external signals with internal developmental and metabolic pathways, enabling coordinated plant-wide outcomes in areas like nutrient allocation, pathogen resistance, and abiotic stress tolerance. Despite these progresses, critical gaps persist, including the criteria for selective sRNA export into transport channels, factors ensuring their integrity during transit, and the decoding mechanisms that dictate their function in target cells. Understanding and utilizing mobile sRNA pathways holds substantial potential for agricultural innovation. Strategies such as CRISPR-mediated editing of microRNA genes, engineering of artificial mobile RNA molecules, targeted RdDM-based epigenome modifications, and grafting techniques for signal delivery could facilitate precise tuning of plant regulatory circuits. However, realizing these benefits demands addressing challenges like unintended off-target impacts, fine-tuned regulation of signal intensity, and comprehensive testing under real-world conditions. In summary, mobile sRNAs serve as a vital link connecting environmental inputs to epigenetic reprogramming in plants. As our comprehension of these molecular interactions evolves, the synergy of RNA sciences, epigenetics, and biotechnological tools advance the creation of resilient crop varieties better equipped to thrive amid escalating changes in climate.
